# Incidence of advanced-stage breast cancer in regular participants of a mammography screening program: a prospective register-based study

**DOI:** 10.1186/s12885-020-6646-5

**Published:** 2020-03-04

**Authors:** Laura Khil, Jan Heidrich, Ina Wellmann, Vanessa Kääb-Sanyal, Stefanie Weigel, Walter Heindel, Hans-Werner Hense, Oliver Heidinger

**Affiliations:** 1 Cancer Registry North Rhine-Westphalia, Gesundheitscampus 10, 44801 Bochum, Germany; 2 Institute for Occupational and Maritime Medicine, University Medical Center Hamburg-Eppendorf, Hamburg, Germany; 3 Institute of Epidemiology and Social Medicine, University of Münster, Münster, Germany; 4 Kooperationsgemeinschaft Mammographie, Berlin, Germany; 5 Institute of Clinical Radiology, University Hospital Münster, Münster, Germany

**Keywords:** Advanced breast cancer - mammography screening programme - incidence rate reduction - population-based cancer registry

## Abstract

**Background:**

The European Guidelines for breast cancer screening suggest that the impact of population-based mammography screening programmes (MSP) may be assessed using the relative reduction in the incidence of advanced breast cancer (ABC, that is, stage UICC II and higher) as a surrogate indicator of screening effectiveness.

**Methods:**

This prospective, population register-based study contained individual data of 1,200,246 women (aged 50–69 years) who attended the initial prevalence screening between 2005 and 2009. Of them, 498,029 women returned for the regular (i.e., within 24 months) first subsequent, and 208,561 for the regular second subsequent incidence screenings. The incidence rate of ABC was calculated for the 24-months period following, but not including, the initial screening by incorporating all interval ABCs and all ABCs detected at the regular first incidence screening; the ABC rate for the second 24-months period was determined in the same way, including ABCs detected in the interval after the first and, respectively, at the second incidence screening. The relative reduction in the ABC incidence was derived by comparing the age-standardized rates in these two periods with an age-standardized reference incidence rate, observed in the target population before the MSP implementation. The strengths and weaknesses of this particular study design were contrasted with a recently published checklist of main methodological problems affecting studies of the effect of MSP on ABC incidence.

**Results:**

The age-standardized ABC incidence rate was 291.6 per 100,000 women for the 24-months period subsequent to the initial screening, and 275.0/100,000 for the 24-months period following the first subsequent screening. Compared to the 2-year incidence of 349.4/100,000 before the start of the MSP, this amounted to a relative reduction of 16.5 and 21.3%, respectively, in the incidence of ABC among regular MSP participants.

**Conclusions:**

The design employed in this study avoids some of the substantial methodological limitations that compromised previous observational studies. Nevertheless, specific limitations prevail that demand a cautious interpretation of the results. Therefore, the study findings, indicating a reduction in ABC for regular MSP participants, need to be followed with respect to potential impacts on breast cancer mortality rates.

## Background

The reduction in the incidence of advanced breast cancer (ABC) is probably the most informative surrogate indicator for the breast cancer mortality decline that may be expected from an organized mammography screening program [1–3]. Numerous observational studies, investigating routine screening programs in various populations, have produced rather inconsistent findings with regard to the changes of ABC incidence observable in the course of the different programs [4–13]. The majority of these studies assessed changes of ABC incidence rates based on aggregated population data because they had no access to individual information on screening history and diagnoses. Of note, as emphasized in a recent literature review [14], in such analyses a mixture of prevalent screenings from a continuous inflow of newly enrolled women with women participating in several consecutive screening rounds, and a screening-exposure time of some participants that is too short to have an effect on the ABC risk, may have compounded the results, with a tendency to underestimate or even obscure actual incidence reductions. In contrast, to the best of our knowledge, only five studies have been published to date that monitored the impact of organized screening programs on ABC incidence using individual-level data on screening exposure. Interestingly, despite large methodological differences (e.g., definition of ABC, definition of screening attender, duration of follow-up, etc.), all five studies observed reductions in the ABC incidence of screening attenders compared to non-attenders [4–8].

The German Mammography Screening Program (MSP) started in October 2005, with the objective to reduce breast cancer associated mortality due to early detection of breast cancer [15]. Population-based cancer registers are an indispensable prerequisite for the identification of interval cancers which are required for the correct calculation of the total ABC incidence among screening participants. The state cancer register for North Rhine-Westphalia (NRW), with 19.9 million inhabitants in 2016 the most populous federal state of Germany, is capable of identifying individual interval breast cancers, being one of only two state registers in Germany performing this task from the time of the beginning of the MSP [16].

In the present report, we analyzed changes in the incidence of ABC, restricted to the group of regular participants of the MSP in NRW. We employed the methods suggested by the European Guidelines [1]. Furthermore, we contrasted the strengths and weaknesses of the present study design with a ten-point checklist of main methodological problems affecting studies of the effect of screening programs on the ABC incidence, recently published by Broeders et al. [14].

## Methods

### The German mammography screening program

The German Mammography Screening Program (MSP) started in October 2005 and has adhered to the European Guidelines for Quality Assurance in Breast Cancer Screening of 2006 [1]. The Kooperationsgemeinschaft Mammographie (German Agency for Mammography Screening), founded by the German Statutory Health Insurances (SHI) and the German Association of SHI Physicians, regularly publishes quality and evaluation reports for the country-wide German MSP which comprises a total of 95 screening units. In these reports, indicators of process quality, participation rates and, in particular, detection rates and stage distributions are cross-sectionally analyzed in detail [15, 17]. All women aged between 50 and 69 years are actively invited every second year to a screening mammography within the systematically organized MSP by a standard invitation letter that contains a suggested examination date and screening unit located most closely to the woman’s home. MSP examinations are performed exclusively in certified screening units. The MSP was fully implemented in North Rhine-Westphalia (NRW) by the beginning of the year 2009 [15], where all women eligible at the start of the program had been invited to participate. The numbers of women in the eligible age range for the program in NRW were approximately 2.2 million and all were invited to the screening by personal letters. About 55% of those invited subsequently participated in the mammography screening program [15, 17].

The state cancer registry of NRW was established in 2005 and completeness of breast cancer registration, using electronic records from obligatory notifications of all newly diagnosed breast cancer cases, was accomplished by 2007 [18]. The NRW cancer registration act authorizes the state cancer registry to process data from all screening units in NRW to evaluate the MSP on the basis of the official Cancer Early Detection Directive in Germany which defines the procedures necessary for the determination of interval cancer rates; identifiers of individual patients were doubly pseudonymized in accordance with procedures laid out in the Cancer Registration Act (Landeskrebsregistergesetz) of the state of North Rhine-Westphalia [19].

In July 2015, records from all MSP participants in NRW were linked with state cancer registry data via a specifically dedicated pseudonymisation procedure [20]. Taking into consideration that the reporting lag between the diagnosis of a breast cancer and its notification and/or registration in the cancer registry may add up to 18 months [21], the study data base for screening participation was closed on December 31st, 2013.

### Study population

**Fig. 1:**
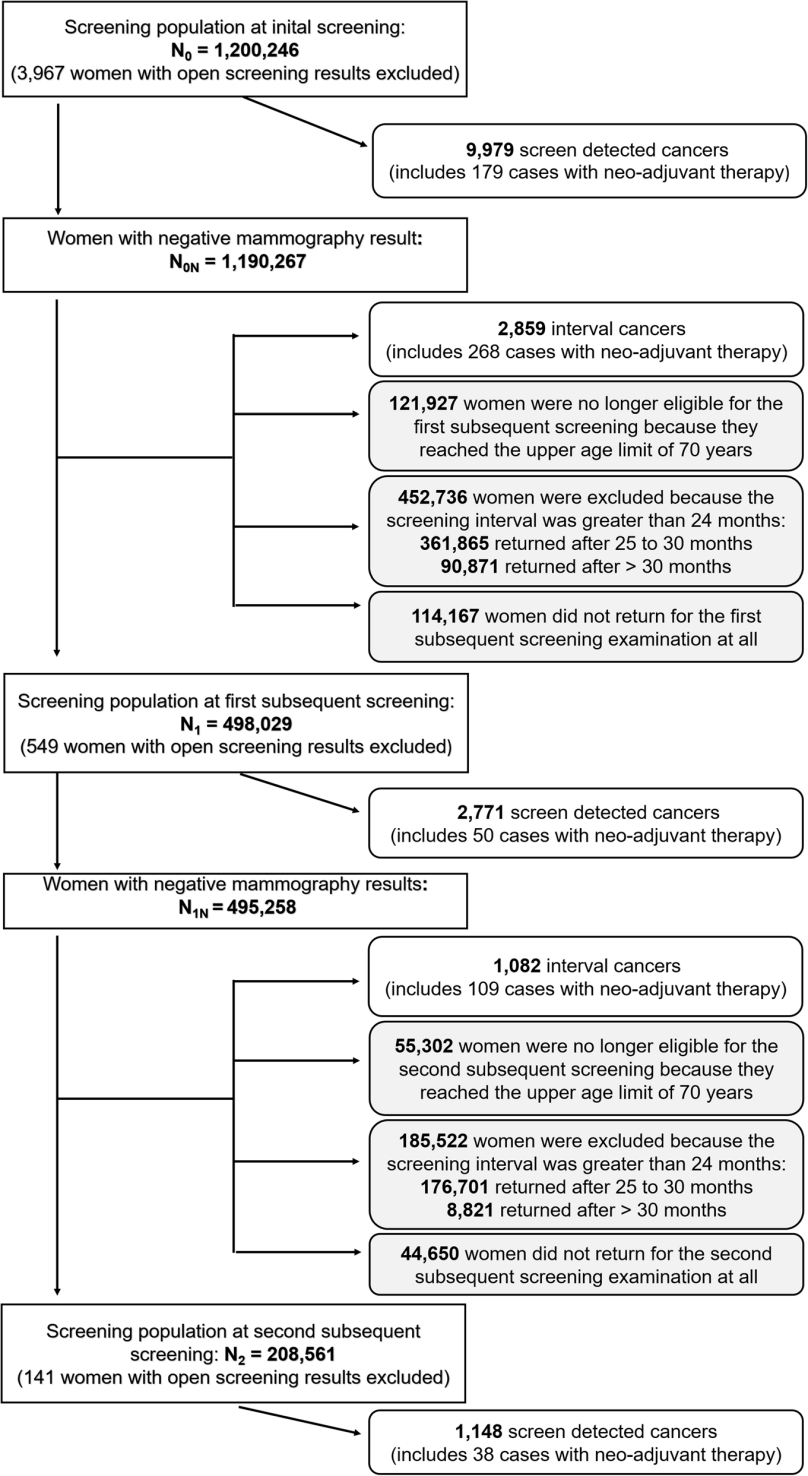
Flow diagram of the study population by screening examination

The study cohort consisted of 1,200,246 eligible screening participants, who attended a first screening mammography (initial screening) during the implementation phase of the program between October 2005 and December 31st, 2009 (Fig. 1). After the initial screening - commonly labelled as prevalence screening - regular MSP participants returned for a first subsequent (incidence) screening examination within the invitation interval of 24 months; likewise, regular participants of the second subsequent (incidence) screening returned within 24 months after their attending the first subsequent screening. Interval cancers were defined as breast cancers occurring within the interval between two regular screenings among women with a prior negative screening mammography. Regular participants of the first incidence screening were women (N_1_ = 498,029) who had no breast cancer detected at the initial prevalence screening nor in the following 24-months interval, and who were still eligible for screening (not older than 69 years), and returned within 24 months after the prevalence screening. Regular participants of the second incidence screening (N_2_ = 208,561) had no breast cancer detected neither at the first subsequent screening nor in the following 24-months interval, were still eligible for screening (not older than 69 years), and they returned for the second subsequent screening within 24 months (Fig. 1). Women with an ‘open’ screening result (commonly women who left the screening program without re-assessment of a conspicuous mammography imaging result) were excluded from the analyses. ‘Death certificate only’ cases (DCO), i.e., breast cancer cases notified to the NRW cancer registry only post mortem by a death certificate, were also excluded prior to data analyses (*n* = 139).

### Definitions and surrogate parameters of effectiveness

The regional *background incidence rate* for breast cancer, defined as the rate of new breast cancers that had been detected in the target population before the initiation of a mammography screening, has been suggested by the European Guidelines as a reference for the rates observed in the course of a screening program [1, 22]. As the statewide cancer registration in NRW started its operation only in 2005, the data from a sub-registry, the cancer registry of the administrative district of Münster, were used to calculate the background incidence. Thus, the background incidence is the incidence in the administrative district of Münster among women aged 50 to 69 years from the years 2000 to 2004. This sub-registry in the north-western part of the state covered about 15% of the total NRW population and has consistently achieved completeness of registration (> 90%) since 1995 [18]. It has been shown that breast cancer data from the Münster registry reflect very closely those from NRW, and these data are also used as reference for the calculation of MSP performance indicators for NRW by the German Agency for Mammography Screening [17]. For the analyses in the present report, the background incidence rate was age-standardized to the distribution of the 5-years age groups in the study cohort. The age-standardized incidence rate was 268.4 per 100,000 women for invasive breast cancer only (ICD-10: C50; 95% CI: 259.0, 275.6), 282.8 (ICD-10: C50 + D05; 95% CI: 274.4, 291.6) per 100,000 women for invasive and in situ breast cancers combined, and 174.7 per 100.000 women for advanced stage breast cancer.

The *breast cancer detection rate* relates the number of invasive and in-situ breast cancers detected by mammography screening to the total number of screening participants. Detection rates and *stage distributions* were excerpted from the data in the MaSc™ documentation software used by each screening unit. The *interval cancer rate* is the proportion of all invasive and in-situ breast cancers newly diagnosed within 24 months post-screening in women who had a negative screening result.

The *incidence rate of advanced stage breast cancer* in regular participants of the first subsequent screening was determined as the sum of the incidence rate of ABC in the interval after a previous negative screening mammography plus the incidence rate of screen-detected ABC in the first incidence screening. Likewise, the ABC incidence rate in regular participants of the second subsequent screening was determined as the sum of the incidence rate of ABC in the interval after a previous negative first incidence screening plus the incidence rate of screen-detected ABC in the first incidence screening. Thus, the incidence of ABC among regular participants was determined for two consecutive 24-months periods.

The *relative reduction of advanced stage breast cancer* was calculated as the ABC incidence, as defined above, in relation to the background incidence rate of ABC according to the following formula suggested by the European guidelines (chapter 1.9.2, page 47) [1]:

1 $$ {\Delta  \mathrm{Inc}}_{\mathrm{ABC}}=\frac{\left[2\ \mathrm{x}\ {\mathrm{BI}}_{\mathrm{ABC}}-\left({\mathrm{ICR}}_{\mathrm{ABC}}+{\mathrm{DR}}_{\mathrm{ABC}}\right)\right]}{2\ \mathrm{x}\ {\mathrm{BI}}_{\mathrm{ABC}}}, $$

where BI_ABC_ is the background incidence rate of ABC, ICR_ABC_ is the ABC interval cancer rate, and DR_ABC_ is the detection rate of ABC in the subsequent screening (following the 24-months period). The multiplication factor 2 for the background incidence BI_ABC_, an annual incidence rate, accommodates the fact that a screening interval lasts 2 years; all incidence rates are presented per 100,000 women.

In the present study, information on tumour size and lymph node involvement were combined to define advanced tumour stages using the UICC-classification [23]. Based on this classification, advanced breast cancers were defined as UICC stage II or higher (UICC II+). UICC II+ was assigned to tumors with a stage of T1N+ (excluding T1N1mic) and all tumors with stage T2 or higher.

Furthermore, of the 17,839 screen and interval detected tumors, 644 (3.6%) had been treated with neo-adjuvant chemotherapy prior to surgery. In order to avoid an underestimation of advanced tumor stages because of a down-staging of breast cancers through neo-adjuvant therapy in clinically advanced tumors, we allocated all tumors with neo-adjuvant treatment to the group of advanced breast cancers.

### Imputation of missing data for tumor stages

Overall, there were 13,898 screen-detected cancers and tumor size (T stage) was missing in 4.2% and N stage was missing in 13.0% of all cases. Of the 3941 interval cancers, tumor size (T stage) was missing in 16.1% and N stage in 19.3%. Likewise, information on T stage was missing in 10.3% and N stage in 16.3% of the 4338 breast cancers constituting the background incidence.

The missing information on T stage and N stage was replaced using multiple imputation by chained equations [24, 25]. Variables potentially related to information on T stage and N stage were selected as predictor variables for the imputation process. These were age at diagnosis, year of diagnosis, morphology (lobular/not lobular), grading, as well as T stage and N stage themselves.

The performance of the imputations was validated in simulation studies employing data of 11,485 women with breast cancer and complete TNM information registered between 2000 and 2012 in the administrative region Münster, and the T stage and N stage and the UICC class were imputed in 20% of cases running 100 simulations. The imputed rate of advanced UICC II+ breast cancers showed an agreement of over 95% with the original staging.

### Statistical analyses

Breast cancer detection rates, interval cancer rates, and the background incidence are given per 100,000 women. To maintain comparability irrespective of differing age composition, all rates and proportions of the first and second subsequent screening examinations as well as the background incidence were standardized to the distribution of the 5-year age groups in the study cohort at the initial screening. We used age at screening for interval and screen-detected cases, and age at diagnosis for the background incidence to classify the age groups. All age-standardized rates as well as age-standardized relative effect measures are provided with 95% confidence limits. All analyses were carried out using SAS™ version 9.4.

## Results

The detection rate for invasive plus in-situ breast cancers was 831.4 per 100,000 women screened at the initial prevalence screening and 537.7 and 518.4 per 100,000, respectively, in the two subsequent incidence screenings (Table 1). Approximately 80% at each screening were invasive cancers, and the proportion of ABC among all screen-detected breast cancers were 32.1, 27.5, and 28.1%, respectively (Table 1).

**Table 1:** Detection rates, numbers and tumor stage of breast cancers among regular participants of the first three screenings of the mammography screening program in North Rhine-Westphalia, 2005–2013

	Initial prevalence screeningN_0_ = 1,200,246	1st subsequent incidence screening^a^ N_1_ = 498,029	2nd subsequent incidence screening^a^ N_2_ = 208,561
Breast cancer detection rate [DR] (per 100,000 screened)	831.4	537.7	518.4
Numbers of invasive cancers	8213	2233	941
Numbers of in-situ cancers	1766	538	207
Invasive cancers / Total cancers screen-detected (%)	82.3	80.6	81.9
Numbers of advanced stage cancers (UICC II+)	3205	762	323
Advanced stage cancers / Total cancers screen-detected (%)^b^	32.1	27.5	28.1

^a^age - standardized to the distribution of the 5-years age groups in the initial screening

^b^based on computations that include *imputed* values (for missing data of size and nodal status) in both, nominator *and* denominator (see text)

The age standardized interval cancer rate (in-situ plus invasive) was 240.2 (83.9 in the first 12 months and 156.3 in the months 13–24) per 100,000 in the interval following the initial screening and 215.3 (77.5 and 137.8, respectively) per 100,000, in the second screening interval (Table 2).

**Table 2:** Numbers and incidence rates of all 24-months interval breast cancers (in-situ plus invasive) after the initial prevalence and the first subsequent incidence screening of the mammography screening program North Rhine-Westphalia, 2005 to 2013

	Number of screened women with negative screening	Number of interval cancers	Interval cancer Incidence rate per 100,000^a^ [95% CI]
After initial screening	1,190,267	2859	240.2 [231.4; 249.0]
After first subsequent screening	495,258	1082	215.3 [202.3; 228.3]

^a^age - standardized to the distribution of the 5-years age groups in the initial screening, 95% CI: 95% confidence interval

The incidence rates of ABC in the 24 months after the initial screening and after the first subsequent screening are shown in Table 3. Using the age-standardized background incidence of UICC II+ breast cancer of 349.4 per 100,000 women in 24 months (see formula (1) above), the relative reduction of UICC II+ breast cancer incidence was 16.5% in the 24-months period after the initial screening and 21.3% in the respective period after the first subsequent screening.

**Table 3:** Numbers and incidence rate of advanced stage breast cancer (UICC II+] in the period after the initial screening (interval ABC plus ABC detected at first subsequent screening] and in the period after the first subsequent screening (interval ABC plus ABC detected at the second subsequent screening], compared with the reference ABC incidence rate and expressed as a relative reduction (in %). Regular participants of the mammography screening program in North Rhine-Westphalia, 2005 to 2013

	UICC II + interval cancers		UICC II + breast cancers detected at screening		Total rate of UICC II+ cancers	Reference incidence rate of UICC II+ cancers	Relative reduction^b^
	n	24-months rate /100,000^a^ [95% CI]	n	24-months rate /100,000^a^ [95% CI]	24-months rate /100,000^a^ [95% CI]	24-months rate /100,000^a^ [95% CI]	(%)
First period after the initial screening	1708	143.5 [136.7; 150.3]	762	148.1 [137.5; 158.8]	291.6 [279.0; 304.2]	349.4 [319.4; 379.6]	16.5 [9.8; 22.8]
Second period after the first subsequent screening	643	127.7 [117.7; 137.7]	323	147.3 [129.7; 165.0]	275.0 [254.7; 295.3]	349.4 [319.4; 379.6]	21.3 [13.5; 28.4]

^a^age - standardized to the distribution of the 5-years age groups in the initial prevalence screening, 95% CI: 95% confidence interval

^b^Formula see methods section

## Discussion

The present study investigated a large cohort of regular screening participants from the mammography screening program in the most populous German state, North Rhine-Westphalia. The breast cancer detection rates and the proportions of invasive cancers were within the ranges recommended by the European guidelines [1]. The proportions of ABC detected at each screening were slightly higher than recommended by the guidelines, which is partly explained by the fact that the present study imputed missing data on tumor stage whereas these are omitted from the nominators when calculating proportions of ABC according to the guidelines. Applying the method proposed in the guidelines, the respective proportion of advanced stage breast cancer were 30.0, 24.9, and 26.3%, respectively.

The decline of ABC rates is commonly invoked as the most important early surrogate indicator of the subsequent reduction of breast cancer mortality in a MSP [1–3]. In a recent meta-analysis of randomized breast cancer screening trials, a reduction of 20% in the incidence of ABC, observed after a trial duration of between five to 10 years, was associated with a 28% reduction in breast cancer mortality [3]. Applying formula (1) from the EU guidelines [1], the incidence of breast cancers of type UICC II+ was about 17% lower in the period after the initial screening, and about 21% after the first subsequent screening in regular MSP participants compared to the reference incidence in the target population immediately before the implementation of the MSP.

Findings from many previous observational studies examining the incidence of ABC in organized mammography screening programs on a population-based level were inconsistent in terms of the incidence reductions observed. In their recent review, Broeders et al. [14] described in detail the methodological problems affecting such studies. They emphasized that, among others, the effects of follow-up time, presence of a prevalence effect, lack of knowledge on the time women were exposed to screening, and the pace of program implementation all interact in a complex way. Many of the limitations outlined by Broeders et al. could potentially be avoided in studies that are able to link individual data on cancer incidence with individual data of screening history. The present study had individual data available for a large cohort of women who regularly responded to three of the biennial invitations of the Germen MSP. It differs from previous study with individual data [4–7] in that it employed – to our knowledge for the first time - the methods suggested by the EU guidelines: the ABC incidence in a cohort of regular screening participants was compared with the reference incidence of ABC in the period prior to the start of the MSP.

In the following, the strengths and limitations of this approach are contrasted with the ten-point checklist suggested by Broeders et al. [14]. The first four methodological problems are related to individual follow-up times, individual exposure time, pace of program implementation and effects of the prevalence screening, all compromising the validity of trend and dynamic population analyses. The present study is able to avoid any of these problems because ABCs detected at the prevalence screening are explicitly excluded from the calculation of incidence rate reductions, the time of screening participation and/or cancer diagnosis is precisely known for each screening participant, and the analyses are restricted to screening participants only. The definition of ABC is clinically relevant, using the UICC categories II and greater, i.e., either lymph node involvement and/or tumor diameter 20 mm or greater. There is, however, a limitation in that the completeness of stage information was missing from a certain percentage of tumors occurring among screened women and in the reference population. The method of imputation had been evaluated in a separate population before [24] and was further assessed by simulations on own data samples with complete stage information (see methods). Furthermore, all cases treated with neoadjuvant therapy were assumed to have been ABC cases. It is supposed that the strategy applied with regard to staging information may not be suspected to underestimate the incidence of ABC in the cohort of regular screening participants.

Furthermore, Broeders at al [14]. also addressed the problem of a stage migration bias due to the introduction of sentinel lymph node biopsy that results into the determination of N1mic cancers, and thus increases the incidence of node positive cancers. However, the exploration and examination of sentinel lymph nodes was standard in German guidelines only in 2004/5. For this reason, prior to 2005, the assessment of micrometastases was not routinely carried out as a standard procedure in pathology laboratories and consequently, micrometastases were not documented in the cancer registry notifications before 2005: the latter were the basis for the calculation of the background ABC incidence rates which are therefore unlikely to be markedly affected by the diagnosis of micrometastases. On the other hand, N1mic are classified as UICC 1b - and not upstaged as ABC (UICC 2+) [26]. The exclusion of N1mic cases after 2005 did therefore not influence the registered incidence of node-positive UICC II+, i.e., ABC cases.

More critical is the use of the background incidence of ABC in the target population before the implementation of the MSP as a reference against which the regular screening participants were compared. This reference assumes that the ABC incidence remained rather stable over the subsequent years. As the ABC incidence in the years before 2005 showed no indication of a temporal trend, we refrained from modelling or extrapolating such a trend. Nevertheless, because the MSP was almost synchronically introduced in the entire country, we had no ‘control group without screening’ available which could have been used to accept or refute the hypothesis of stable secular ABC trends. Of note, analyses of ABC incidence trends in the adjacent age groups (40–49 and 70–79 years) seem to indicate that the stability hypothesis is sustainable [11] and that the ABC rates in the population before the introduction of the MSP is a reasonable reference. Furthermore, incidence data from the period before the introduction of the MSP were available only for the administrative district of Münster, which covers only about 15% of the NRW population. Additional analyses confirmed, however, that the breast cancer incidence rates after 2005 were very similar in the Münster District and NRW, indicating that it is unlikely that the application of the Münster rates invalidate the reference values of this study. Moreover, the reference incidence rates were estimated using a population not yet offered an organized screening program, thus including a mix of women who would potentially have participated in such a program, if offered, and of women who would not have participated. If these two groups differed in their ABC risks, this could probably affect the validity of the comparison, in particular, if the risk was a priori lower in potential screening participants (one sort of healthy screenee bias [27]). Without a screening program, such a lower incidence of ABC in potential screening participants could be due either to them having a set of risk factors that differentially leads to less rapidly growing tumors. However, Puliti et al. [4] found that although socioeconomic status, as an indicator of health – related lifestyle habits, was associated with screening participation, it did not affect the estimated relative reduction in advanced stage breast cancer. Alternatively, a generally higher compliance with and a better adherence to preventive health care offers could result in a generally earlier breast cancer diagnosis, e.g., by opportunistic screening offers. To our knowledge there is no evidence supporting the first, pathophysiological assumption, while the latter would require that a rather massive coverage of women with opportunistic screening mammographies had been in place before implementation of the MSP in order to be effectively able to lower their ABC rates. Marked rises in the ABC rates during the prevalence round of the German MSP render such a hypothesis rather unlikely. In fact, wide-spread opportunistic screening would have tended to attenuate the observed extent of ABC reduction.

Another aspect deserves attention: a cut-off of 24 months defined ‘regular’ participation. This approach has the advantage that it uses the conventional definition of the period for interval cancer occurrence, but it also allows a straight-forward comparison with the reference rates with the use of twice the annual background population incidence rate (see formula 1) [1]. Restricting the definition to 24 months ignores, however, that a substantial proportion of women returned to the screening later than 24 months (Fig. 1) which is, among others, due to organizational aspects (the MSP invitation is required to be mailed within 22 to 26 months after the precedent invitation) or women’s preferences (e.g. work, vacation, other commitments, etc.). The reduction in ABC rates reported here holds therefore strictly only for a 24-months screening interval.

One point not addressed by Broeders et al. [14] is the increasing use of neo-adjuvant therapy, especially in ABC cases. In order to avoid an underestimation of advanced tumor stages due to downstaging after neo-adjuvant therapy, we allocated all tumors with information on neo-adjuvant therapy prior to surgery to the group of advanced breast cancers. This may have marginally inflated the incidence of ABC in the later years of the MSP and thus have attenuated the observed relative reductions.

## Conclusions

In conclusion, this study used a methodological approach that avoids many of the shortcomings of previous reports. However, this study still contains certain limitations that have to be considered when interpreting the results, such as the historical background incidence, the lack of information on the extent of opportunistic screening before the implementation of the MSP, and the missing stage information before neo-adjuvant treatment. Keeping these limitations in mind, this study suggests that the MSP in NRW is able to lower the incidence of ABC among regular participants by about 21% after a screening period of 4 years when compared to the reference ABC incidence observed in the target population before implementation of the program.

## Data Availability

The datasets generated and/or analysed during the current study are not publicly available due to local data protection policies. However, the data can be accessed on-site at the Cancer Registry North Rhine-Westphalia upon reasonable request.

## Abbreviations

ABC: Advanced breast cancer
BI: Background incidence
CI: Confidence interval
DR: Detection rate
MSP: Mammography screeing programme
NRW: North Rhine-Westphalia
